# ChatGPT-4-Based Automated Preliminary Clinical Reporting in Myocardial Perfusion Imaging: A Pilot Evaluation

**DOI:** 10.3390/diagnostics16193110

**Published:** 2026-09-24

**Authors:** Mutlay Keskin, Ece Oğuz

**Affiliations:** 1 Department of Nuclear Medicine, Mersin City Training and Research Hospital, 33240 Mersin, Turkey; 2 Department of Family Medicine, Mersin City Training and Research Hospital, 33240 Mersin, Turkey

**Keywords:** ChatGPT-4, myocardial perfusion scintigraphy, artificial intelligence, automated reporting, nuclear medicine

## Abstract

**Background/Objectives:** Myocardial perfusion scintigraphy (MPS) is a cornerstone non-invasive imaging modality for assessing myocardial ischemia and infarction. This pilot study aimed to evaluate the feasibility of using ChatGPT-4 for automated preliminary clinical reporting in MPS and to identify specific scenarios in which large language model (LLM) performance is robust versus inadequate. **Methods:** A comparative analysis was conducted using 30 consecutive de-identified MPS cases spanning a broad spectrum of clinical scenarios. Structured clinical data were input into ChatGPT-4 to generate AI-based preliminary reports, which were then compared with reports prepared by two experienced nuclear medicine physicians. Reports were independently evaluated using four criteria—clinical accuracy, report structure, terminological appropriateness, and overall comprehensibility—scored on a 5-point Likert scale. Inter-observer agreement was assessed using Cohen’s kappa. **Results:** ChatGPT-4 demonstrated strong performance in report structure (median 5), terminological appropriateness (median 4), and overall comprehensibility (median 5), consistently producing well-organized and coherent reports. However, clinical accuracy was significantly lower compared with physician reports (median 4 vs. 5; *p* = 0.002; effect size *r* = 0.52), particularly in complex cases such as multivessel ischemia and mixed pathologies, where outputs were occasionally superficial or lacked specificity. Inter-observer agreement between evaluating physicians was substantial (Cohen’s κ = 0.78; 95% CI: 0.64–0.92). **Conclusions:** ChatGPT-4 shows promise as a supportive tool for preliminary MPS reporting and medical education, but its limitations in higher-order clinical reasoning necessitate careful human oversight. These findings are preliminary and require validation in adequately powered, multicenter studies before any clinical implementation.

## 1. Introduction

Myocardial perfusion scintigraphy (MPS) with single-photon emission computed tomography (SPECT) remains a cornerstone non-invasive imaging modality for the diagnosis, risk stratification, and management of coronary artery disease (CAD) [1,2]. Accurate and timely interpretation of MPS studies is essential for guiding therapeutic decisions; however, the translation of raw imaging data into structured, clinically actionable reports is a cognitively demanding process that contributes substantially to physician workload, particularly in high-volume nuclear cardiology centers.

In recent years, artificial intelligence (AI) has been increasingly integrated into medical imaging workflows, with applications ranging from automated image segmentation and quantitative perfusion analysis to computer-aided detection systems [3]. Concurrently, large language models (LLMs) such as OpenAI’s GPT-4 have demonstrated remarkable proficiency in natural language understanding and generation across diverse domains, including medicine [4,5,6,7]. Emerging studies have explored LLM applications in radiology report simplification, differential diagnosis generation, and clinical documentation [8,9]. Notably, recent systematic reviews have highlighted that while LLMs show promise in radiology reporting, limitations in diagnostic accuracy, hallucinations, and inconsistencies necessitate rigorous human oversight [10].

In nuclear medicine specifically, LLMs have begun to demonstrate value in interpretative and workflow tasks. For example, retrieval-augmented generation (RAG) models integrated with PET imaging report databases have achieved over 84% success rates in retrieving relevant similar cases and significantly improved diagnostic appropriateness scores compared to standalone LLMs [11]. Additionally, domain-specific models such as fine-tuned RoBERTa-base for Deauville scoring have demonstrated high accuracy in lymphoma assessments [12]. However, the application of general-purpose LLMs such as ChatGPT-4 to myocardial perfusion reporting—where nuanced integration of stress physiology, perfusion patterns, and coronary territory correlation is required—remains largely unexplored.

Despite these advances, several critical gaps remain in the literature. First, the majority of LLM studies in medical imaging have focused on radiology rather than nuclear cardiology, leaving the feasibility of LLM-assisted MPS reporting largely unexplored. Second, existing evaluations often rely on synthetic or simplified clinical vignettes rather than real structured patient data derived from actual imaging studies. Third, there is a paucity of data comparing LLM-generated reports directly against expert physician reports using validated, domain-specific quality criteria. Recent evidence indicates that LLMs achieve moderate to substantial agreement with expert protocols for mandatory imaging sequences but only fair agreement for optional or complex scenarios [13], suggesting that performance may be task-dependent.

Against this background, the present pilot study was designed to evaluate whether ChatGPT-4 can generate clinically useful preliminary reports from real, structured MPS data. Specifically, we aimed to (i) assess the clinical accuracy, terminological appropriateness, structural quality, and comprehensibility of AI-generated reports compared to those prepared by experienced nuclear medicine physicians; (ii) identify specific scenarios in which LLM performance is robust versus inadequate; and (iii) discuss the implications for clinical workflow integration and medical education. We hypothesized that ChatGPT-4 would demonstrate high structural and linguistic competence but exhibit limitations in complex clinical reasoning requiring integrated pathophysiological interpretation.

## 2. Materials and Methods

### 2.1. Study Design

This retrospective observational pilot study aimed to evaluate the feasibility and performance of AI-assisted preliminary clinical reporting in MPS. A comparative analysis was conducted between preliminary clinical reports generated by ChatGPT-4, SPSS, R and reports prepared by experienced nuclear medicine physicians using real patient MPS data. The primary objective was to assess AI-generated reports in terms of clinical accuracy, report structure, terminological appropriateness, and overall comprehensibility. Thirty consecutive de-identified MPS cases were selected to represent a broad spectrum of commonly encountered clinical scenarios in nuclear cardiology, a sample larger than those of prior feasibility studies, though still modest and appropriate only for exploratory, hypothesis-generating evaluation. All statistical analyses were conducted as exploratory evaluations, and findings are presented as hypothesis-generating rather than confirmatory.

### 2.2. Case Selection and Preparation

Thirty consecutive MPS cases were selected from de-identified patient data collected retrospectively over a 12-month period between January 2024 and December 2024. The specific data collection timeframe is explicitly reported here to ensure full transparency and reproducibility. All patient identifiers were removed to ensure complete anonymity. The selected cases were reviewed to confirm they reflected a broad spectrum of clinical scenarios encountered in routine nuclear cardiology practice (Table 1).

Case preparation was performed by a physician who did not participate in the subsequent evaluation or scoring process, minimizing potential bias. All cases consisted of myocardial perfusion SPECT studies acquired on a dual-head gamma camera (Discovery NM630, GE HealthCare, Waukesha, WI, USA) using technetium-99m sestamibi (Curium, Petten, Netherlands).

To ensure consistency across cases, a standardized data extraction template was developed. For each patient, the following structured data fields were compiled from the imaging workstation:

- Demographics and Clinical Context: Age, sex, body mass index (BMI), clinical indication for MPS (e.g., “exertional chest pain,” “preoperative risk stratification”), and relevant cardiac history (prior MI, revascularization, known CAD).
- Stress Protocol: Type of stress (exercise treadmill Bruce protocol vs. pharmacologic with adenosine (Adozin, Vem İlaç, Istanbul, Turkey)), peak heart rate, blood pressure response, and symptoms during stress.
- Perfusion Findings (Rest): Segmental perfusion status for each vascular territory (left anterior descending [LAD], left circumflex [LCx], right coronary artery [RCA]) using a 17-segment model, categorized as normal, mildly reduced, moderately reduced, or severely reduced.
- Perfusion Findings (Stress): Segmental perfusion status under stress with explicit notation of reversibility (reversible, fixed, partially reversible) for each abnormal segment.
- Left Ventricular Function: Ejection fraction (%), end-diastolic volume (mL), end-systolic volume (mL), regional wall motion (normal, hypokinetic, akinetic, dyskinetic), and wall-thickening assessment.
- Technical Quality Indicators: Presence of motion artifacts, attenuation artifacts (breast-, diaphragmatic-, or body habitus-related), submaximal stress achievement, or tracer uptake issues.

All data were extracted by a single nuclear medicine physician and verified against the official structured report and DICOM headers. No imaging files, raw projection data, or quantitative polar maps were provided to the AI.

### 2.3. ChatGPT-4 Reporting Procedure

The structured clinical data for each case were input into the OpenAI-developed ChatGPT-4 interface. ChatGPT-4 did not directly access or interpret imaging files; all findings were provided as structured textual descriptions derived from physician interpretation.

A standardized prompt was applied for all cases:

*“Based on the myocardial perfusion scintigraphy findings provided below, generate a preliminary clinical report. The report should be written as if prepared by a nuclear medicine specialist, using appropriate medical terminology, in a structured format, and intended for nuclear medicine physicians and cardiologists.”*

Generated reports were recorded verbatim. In 28 of 30 cases (93.3%), the reports included standardized sections titled Findings, Interpretation, and Conclusion.

#### 2.3.1. Technical Specifications and Reproducibility

All AI-generated reports were produced using the GPT-4 model (OpenAI, San Francisco, CA, USA), accessed via the ChatGPT web interface between January 2026 and February 2026. The specific model version was GPT-4 (gpt-4-1106-preview/GPT-4 Turbo, as indicated by the platform at the time of access). No custom fine-tuning or domain-specific adaptation was performed; the model was used in its default configuration.

The standardized prompt was entered identically for each case without modification. No temperature, top-p, or token limit adjustments were manually configured; the model operated under its default inference parameters (temperature approximately 0.7–1.0, max tokens dynamically allocated). To ensure reproducibility, the exact prompt text, input data structure, and system response timestamps were documented for each case. It should be noted that LLM outputs may exhibit minor variability across sessions due to the probabilistic nature of token generation; however, in this study, all reports were generated within a single session to maximize consistency.

#### 2.3.2. Input Feature Construction

For each case, the structured data described in Section 2.2 was formatted into a standardized textual block using a consistent hierarchy: (1) patient demographics and indication; (2) stress test details; (3) rest and stress perfusion findings by coronary territory; (4) LV function parameters; and (5) technical quality notes. This format was designed to mimic the information density of a typical nuclear cardiology handoff without including interpretive conclusions.

An example of the input structure for Case 2 (mild anterior ischemia) is provided below.

Input Example (Case 2):

*“Patient: male. Indication: Atypical chest pain. Stress protocol: Exercise treadmill Bruce protocol, achieved 9 METs, peak HR 162 bpm (85% max), no angina, no ST depression. Rest perfusion: Normal homogeneous uptake across all segments. Stress perfusion: Mildly reduced uptake in the mid-anterior and anteroseptal walls (segments 7, 8, 13, 14) with complete reversibility at rest. No fixed defects. LVEF: 58% (rest), 62% (stress). Wall motion: Normal. Technical quality: Good; no significant motion or attenuation artifacts.”*

This structured input was then appended to the standardized prompt described above to generate the final query submitted to ChatGPT-4.

#### 2.3.3. Processing Mechanism of Structured Clinical Data by the LLM

ChatGPT-4 processes textual input through a transformer-based architecture utilizing multi-head self-attention mechanisms. When structured clinical data are submitted, the model first tokenizes the input into subword units, embeds these tokens in a high-dimensional vector space, and processes them through multiple layers of attention to identify semantic relationships between clinical entities (e.g., associating “reversible defect” with “ischemia” and “anterior wall” with “LAD territory”).

The model generates output tokens autoregressively, predicting the most probable next token based on the input context and its training distribution, which includes vast corpora of medical literature, textbooks, and clinical guidelines. It is important to emphasize that ChatGPT-4 does not perform logical deduction or pathophysiological simulation; rather, it generates linguistically coherent responses by recognizing statistical patterns between input features and typical nuclear cardiology report structures. This distinction explains why the model excels at structural and terminological tasks but may falter in cases requiring novel clinical inference beyond its training distribution.

### 2.4. Selection of Evaluation Criteria

Four evaluation criteria—clinical accuracy, report structure, terminological appropriateness, and overall comprehensibility—were selected based on established standards for nuclear medicine reporting quality. These criteria are commonly used in studies assessing medical imaging report quality and AI-assisted reporting tools (Table 2).

These criteria were chosen to reflect key aspects of report quality that impact both patient care and workflow efficiency. Each criterion was scored using a 5-point Likert scale (1 = Poor, 2 = Fair, 3 = Moderate, 4 = Good, 5 = Excellent) to ensure reproducible and standardized assessment.

### 2.5. Physician Comparison and Evaluation Process

The preliminary reports generated by ChatGPT-4 were independently compared with reports prepared by two senior nuclear medicine physicians (with >10 years of experience in nuclear cardiology), who were considered the reference standard for clinical interpretation in this study. Each report was evaluated based on the predefined criteria described above.

The evaluation was not blinded, and the origin of the reports was not masked: both evaluators were aware that they were scoring reports generated by ChatGPT-4. Each physician independently scored the AI-generated report of every case against his or her own reference report using the predefined criteria, without access to the other evaluator’s scores. All cases had been prepared by a separate physician who did not participate in scoring. Because the two evaluators had also authored the reference reports, an assessor bias in favor of the physician reports cannot be excluded; this limitation is explicitly acknowledged in the Section 4.6.

Scores from both evaluators were summarized using medians for standardized comparison. This approach allowed AI-generated reports to be assessed against the aggregated expert evaluations, which served as the reference standard based on expert consensus.

### 2.6. Data Analysis

Given the ordinal nature of Likert scale data, results are presented as medians with interquartile ranges (IQR). Comparisons between ChatGPT-4 and physician reports were performed using the Wilcoxon signed-rank test for paired ordinal data. Effect size was calculated as the rankbiserial correlation coefficient (r). Inter-observer agreement between the two evaluating physicians was assessed using Cohen’s kappa (κ) with 95% confidence intervals, interpreted according to Landis and Koch criteria (κ < 0.20: slight; 0.21–0.40: fair; 0.41–0.60: moderate; 0.61–0.80: substantial; 0.81–1.00: almost perfect).

Qualitative feedback from the evaluating physicians regarding the strengths and limitations of ChatGPT-4 reports was collected and thematically analyzed. Descriptive statistics were used to summarize performance across case categories.

All statistical analyses were performed using SPSS version 28.0 (IBM Corp., Armonk, NY, USA) and R version 4.3.1 (R Foundation for Statistical Computing, Vienna, Austria). A two-tailed *p*-value < 0.05 was considered statistically significant.

### 2.7. Ethical Considerations

All MPS studies were performed as part of routine clinical care in accordance with institutional nuclear protocols, and all patients provided written informed consent for the imaging procedure at the time of examination using the standard institutional consent form. The retrospective analysis of fully de-identified data was conducted in accordance with the Declaration of Helsinki and was approved by the Ethics Committee of the Mersin City Training and Research Hospital (approval date: 20 November 2025; approval code: 11.20.2025-22). Data were accessed and analyzed only after ethics approval was granted.The requirement for informed consent for this retrospective analysis was waived by the Ethics Committee because the analysis involved only fully de-identified data.

## 3. Results

### 3.1. Overall Performance Evaluation

Thirty preliminary MPS reports generated by ChatGPT-4 were compared with reports prepared by two physicians and scored based on four distinct criteria. Results are presented as median scores across evaluators. ChatGPT-4 received particularly high median scores in terms of report structure and overall comprehensibility. In terms of clinical accuracy, however, certain cases revealed incomplete or superficial interpretations, resulting in comparatively lower median scores than physician-generated reports. Regarding terminological appropriateness, ChatGPT-4 demonstrated consistent use of medical language overall, though it occasionally lacked specificity in technical expressions (e.g., using “temporary reduction in blood flow” instead of the more precise “reversible defect”; Table 3). Given the ordinal nature of the scoring system, all results are reported as medians.

Physician scores demonstrated high consistency across cases and criteria, with inter-observer agreement assessed as substantial (Cohen’s κ = 0.78; 95% CI: 0.64–0.92).

### 3.2. Performance by Clinical Scenario

Subgroup analysis by case category revealed differential AI performance across clinical scenarios (Table 4). ChatGPT-4 performed most robustly in normal perfusion cases and single-vessel ischemia, where clinical patterns are well-represented in training data. Performance was lowest in multivessel ischemia and mixed/complex pathology cases, where integrated pathophysiological reasoning was required.

### 3.3. Qualitative Observations

ChatGPT-4 tended to simplify language, particularly in the “findings explanation” and “clinical impression” sections. While this improved readability, it occasionally resulted in the omission of technical nuances.

The reports consistently maintained a structured format, with clear sections such as Findings, Interpretation, and Conclusion. In 28 of 30 cases (93.3%), the AI-generated reports included all three required sections without prompting.

Physicians considered ChatGPT-4-generated reports suitable as preliminary assessments but emphasized that final clinical reporting should always involve human oversight. For example, in cases involving reversible perfusion defects, physician reports explicitly described the pattern as suggestive of ischemia in a specific coronary territory, whereas the AI-generated report described the finding more generally as a “temporary reduction in blood flow,” without further anatomical or clinical specification in 8 of 14 ischemic cases (57.1%).

### 3.4. Statistical Results

A Wilcoxon signed-rank test indicated a statistically significant difference between ChatGPT-4 and physician reports in the criterion of clinical accuracy (median 4 vs. 5; *p* = 0.002; effect size *r* = 0.52, indicating a medium-to-large effect). No statistically significant differences were observed for terminological appropriateness (*p* = 0.18), report structure (*p* = 0.11), or overall comprehensibility (*p* = 0.42). Inter-observer agreement between the two evaluating physicians was substantial (Cohen’s κ = 0.78; 95% CI: 0.64–0.92).

## 4. Discussion

In this study, the potential of ChatGPT-4, an LLM-based artificial intelligence application, to automatically generate preliminary clinical reports from real de-identified patient MPS data was evaluated. Analyses conducted on 30 real patient cases demonstrated that ChatGPT-4 exhibited high performance in producing comprehensible, well-structured, and terminologically consistent reports; however, it lagged behind physicians in terms of clinical accuracy and depth of interpretation, particularly in complex diagnostic scenarios.

### 4.1. Clinical Accuracy and Adequacy of Medical Interpretation

According to the study findings, ChatGPT-4 received lower scores in clinical accuracy, particularly in complex or interpretation-dependent cases (e.g., multivessel ischemia, artifact differentiation, mixed pathology). These findings suggest that while the model performs well in basic clinical pattern recognition, it remains limited in cases requiring advanced clinical reasoning and pathophysiological correlation.

The lower clinical accuracy scores observed in complex cases stem from several inherent limitations of LLM architecture. First, ChatGPT-4 lacks access to spatial and temporal imaging data; it cannot evaluate raw SPECT projections, gated motion frames, or attenuation maps. Second, the model operates without patient-specific clinical context (e.g., prior cardiac catheterization results, biomarker trends, or exercise tolerance test data), which experienced physicians integrate subconsciously during interpretation. Third, LLMs generate responses based on statistical patterns in training data rather than causal pathophysiological reasoning. For instance, when presented with “widespread reversible defects,” the model described “reduced perfusion in multiple regions” but failed to infer the hemodynamic significance of balanced ischemia or to prioritize multivessel disease in the differential diagnosis. This reflects a fundamental constraint: LLMs excel at pattern matching and linguistic structuring but lack the clinical intuition required to weigh competing diagnostic probabilities in ambiguous scenarios.

In multivessel ischemia cases, the model failed to suggest the possibility of “multivessel disease” in 3 of 4 cases, revealing an inability to infer latent clinical meanings. Similar limitations have been reported in prior research on radiology reporting. Jeblick et al. found that ChatGPT-4 possesses a strong capacity to articulate basic radiological findings, yet continues to demonstrate weaknesses in contexts requiring “clinical decision support” [14]. Recent systematic reviews have confirmed that while LLMs show promise in radiology reporting, hallucinations, misdiagnoses, and inconsistencies remain prevalent across all evaluated models [10].

### 4.2. Linguistic and Structural Performance

ChatGPT-4’s consistently high scores in report structuring and language use reflect its mastery of linguistic rules. Reports regularly employed standard headings such as Findings, Assessment, and Conclusion, and presented statements clearly and coherently. These characteristics suggest that ChatGPT-4 could serve as an educational tool for medical students, helping them gain experience in structured report writing [15]. Furthermore, the model’s ability to explain complex terminology in simpler, patient-friendly language suggests potential utility in patient communication, although this aspect was beyond the scope of the present study.

### 4.3. Terminological Consistency and Accuracy of Medical Expression

Terminological analysis revealed that ChatGPT-4 generally employed accurate and established medical terminology. Expressions such as “reversible ischemia” and “fixed perfusion defect” were correctly used, indicating that the model has been sufficiently exposed to medical language during training. Nevertheless, in some cases, more generic and less technically detailed expressions were observed, underscoring that the model does not operate according to formal clinical guidelines or integrate with evidence-based decision frameworks. Such limitations underscore the importance of using AI systems solely as decision-support tools in clinical practice to ensure patient safety.

### 4.4. Comparison with Existing Literature on LLM-Assisted Medical Reporting

Our findings align with and extend prior investigations on LLM applications in medical imaging. Jeblick et al. demonstrated that ChatGPT-4 could simplify radiology reports while preserving core clinical information, although they similarly noted limitations in nuanced clinical reasoning [14]. Hirosawa et al. reported that ChatGPT-generated differential diagnoses achieved moderate accuracy for complex clinical vignettes but struggled with multi-system pathologies requiring integrated pathophysiological reasoning [16]. Biswas highlighted the potential of ChatGPT in medical writing while cautioning against over-reliance in contexts requiring expert clinical judgment [9].

Recent studies on LLM-assisted cardiovascular imaging have yielded comparable results. Licu et al. evaluated multiple LLMs (including ChatGPT 4.1) for CMR protocol generation and found moderate overall agreement with society guidelines (Cohen’s κ = 0.52 for ChatGPT 4.1), with substantial agreement for mandatory sequences (Fleiss κ = 0.71) but only fair agreement for optional or complex scenarios [13]. This pattern—strong performance on standardized tasks but weaker performance on nuanced clinical decisions—mirrors our findings in MPS reporting.

In the specific domain of nuclear medicine, automated reporting has traditionally relied on rule-based algorithms and machine learning classifiers applied directly to imaging data (e.g., automated SPECT quantification software) [3]. Unlike these specialized tools, ChatGPT-4 operates purely on structured textual input without direct image interpretation capabilities. This represents a fundamentally different paradigm: whereas CAD systems analyze pixel-level perfusion data, LLMs synthesize clinical narratives from human-interpreted findings. Consequently, LLM-based reporting should be viewed as complementary to, rather than a replacement for, image-based AI tools. Future hybrid architectures integrating automated quantification software with LLM-based narrative generation may offer the optimal balance of diagnostic precision and linguistic coherence.

Importantly, retrieval-augmented generation (RAG) approaches have shown promise in nuclear medicine workflows. Choi et al. developed a RAG-LLM integrated with over 211,000 PET imaging reports, achieving an 84.1% success rate in retrieving relevant, similar cases and significantly higher diagnostic appropriateness scores compared to standalone LLMs [11]. This suggests that integrating LLMs with institutional knowledge bases may partially mitigate the clinical reasoning limitations observed in our study.

### 4.5. Educational and Workflow Implications

While ChatGPT-4 offers general natural language processing capabilities, dedicated AI/ML tools for nuclear cardiology (e.g., automated quantification software for SPECT/PET perfusion analysis, CAD systems, or ML-based interpretation assistants) may provide more precise diagnostic outputs by directly analyzing imaging data [3]. Integrating ChatGPT-4 outputs with these specialized tools could enhance report accuracy while preserving efficiency, particularly in high-volume clinical settings.

The study results indicate that ChatGPT-4 can be particularly useful as an educational tool for medical students and residents. Students can engage in comparative report writing exercises using MPS data and ChatGPT-4 outputs, thereby improving both their medical terminology and clinical reasoning skills [15,17]. Moreover, in busy clinical environments, hybrid systems in which ChatGPT-4 generates preliminary draft reports later reviewed and edited by specialists could enhance efficiency and time management, especially in large hospitals with heavy reporting workloads. Importantly, the model should be positioned as assistive rather than autonomous, ensuring final clinical decisions remain with qualified physicians.

### 4.6. Limitations

Several limitations should be acknowledged. First, although the 30-case cohort is larger than the very small samples typical of early feasibility studies, it remains insufficient for definitive generalization and was not intended to provide confirmatory statistical power. Second, the evaluation was limited to two physicians from a single institution; inter-institutional variability in reporting practices was not captured. Third, the model was not fine-tuned specifically with nuclear cardiology data, and it did not have direct access to imaging files; its performance was thus limited to structured textual input. Fourth, the use of a Likert-scale-based subjective evaluation may introduce inter-observer variability, although efforts were made to minimize this through independent assessment by two physicians blinded to each other’s scores (substantial inter-observer agreement, κ = 0.78). Fifth, the evaluation was not masked: the two evaluators were aware that the reports they scored had been generated by ChatGPT-4 and compared them with their own reference reports, which may have introduced assessor bias.

Finally, as LLM technology evolves rapidly, newer models (e.g., GPT-4o, GPT-4.1, multimodal variants) may demonstrate improved clinical reasoning capabilities. The present findings should be interpreted in the context of the specific model version evaluated (GPT-4 Turbo, January–February 2026).

### 4.7. Clinical and Ethical Implications

Integrating large language models like ChatGPT-4 directly into clinical reporting workflows raises substantial concerns regarding medical ethics, patient safety, and legal accountability. Clinical decisions based on inaccurate or incomplete AI-generated reports could lead to serious adverse outcomes. Therefore, such tools must be strictly assistive, with final clinical decisions made by qualified specialists [18,19]. Furthermore, if the model interacts with patient data, explicit policies and regulatory oversight must be established to ensure data privacy and security. The findings of this study support the emerging consensus that LLMs should be deployed as “copilots” rather than autonomous agents in clinical documentation workflows [10].

## 5. Conclusions

ChatGPT-4 demonstrated strong linguistic fluency and structural consistency in generating preliminary MPS reports across a 30-case cohort, highlighting its potential as a supportive tool in medical education and reporting workflows. However, its limitations in higher-order clinical reasoning, particularly in complex diagnostic scenarios such as multivessel ischemia and artifact differentiation, underscore the necessity of human oversight. Future integration should focus on hybrid models combining AI-generated drafts with expert validation, supported by robust ethical and regulatory frameworks. These findings should be considered preliminary and require validation in adequately powered studies before any clinical implementation. Larger multicenter studies are warranted to validate these findings and establish standardized protocols for LLM-assisted nuclear cardiology reporting.

## Figures and Tables

**Table 1:** Distribution of 30 MPS cases by clinical scenario.

Case Category	*n*	Description	Key Features
Normal perfusion	6	No perfusion defects	Symmetric uptake, normal LVEF
Single-vessel ischemia (LAD)	6	Reversible defect in LAD territory	Anterior/anteroseptal reversibility
Single-vessel ischemia (RCA/LCx)	4	Reversible defect in RCA or LCx territory	Inferior/inferolateral or lateral reversibility
Multivessel ischemia	4	Reversible defects in ≥2 territories	Widespread reversible perfusion abnormalities
Old myocardial infarction	4	Fixed perfusion defects	Fixed defects with wall motion abnormalities
Attenuation/technical artifact	4	Breast, diaphragmatic, or motion artifact	Basal inferior or anterior hypoperfusion with artifact suspicion
Mixed/complex pathology	2	Combination of ischemia + infarct or artifact + ischemia	Multiple competing findings

**Table 2:** Evaluation criteria used for report assessment.

Evaluation Criterion	Description
Clinical Accuracy	Accuracy in interpreting findings and disease characterization
Terminological Appropriateness	Use of relevant and meaningful medical terminology
Report Structure	Presentation of the report in a professional and organized format
Overall Comprehensibility	Clarity, simplicity, and logical flow of language

**Table 3:** Scores of ChatGPT-4 and Physician Reports Based on Evaluation Criteria (5-point Likert Scale, *n* = 30).

Evaluation Criteria	ChatGPT-4 Median (IQR)	Physician Median (IQR)	*p*-Value	Effect Size (*r*)
Clinical Accuracy	4 (3-4)	5 (4-5)	0.002	0.52
Terminological Appropriateness	4 (4-5)	5 (4-5)	0.18	0.22
Report Structure	5 (4-5)	5 (5-5)	0.11	0.28
Overall Comprehensibility	5 (4-5)	5 (4-5)	0.42	0.14

**Table 4:** ChatGPT-4 Clinical Accuracy Scores by Case Category (Median, IQR).

Case Category	*n*	Clinical Accuracy Median (IQR)	Physician Median (IQR)
Normal perfusion	6	5 (4-5)	5 (5-5)
Single-vessel ischemia (LAD)	6	4 (4-5)	5 (5-5)
Single-vessel ischemia (RCA/LCx)	4	4 (4-5)	5 (5-5)
Multivessel ischemia	4	3 (3-4)	5 (4-5)
Old myocardial infarction	4	4 (3-4)	5 (5-5)
Attenuation/technical artifact	4	3 (3-4)	5 (4-5)
Mixed/complex pathology	2	3 (3-3)	4 (4-5)

## Data Availability

The data presented in this study are available upon reasonable request from the corresponding author. The data are not publicly available due to ethical and privacy restrictions involving human participants.
